# The Importance of Marine Omega-3s for Brain Development and the Prevention and Treatment of Behavior, Mood, and Other Brain Disorders

**DOI:** 10.3390/nu12082333

**Published:** 2020-08-04

**Authors:** James J. DiNicolantonio, James H. O’Keefe

**Affiliations:** Saint Luke’s Mid America Heart Institute, Kansas City, MO 64111, USA; jokeefe@saintlukeskc.org

**Keywords:** omega-3, fish oil, brain, mood, attention, depression

## Abstract

Most of the global population is deficient in long-chain marine omega-3s. In particular, docosahexaenoic acid (DHA), a long-chain omega-3 fatty acid, is important for brain and eye development. Additionally, DHA plays a significant role in mental health throughout early childhood and even into adulthood. In the brain, DHA is important for cellular membrane fluidity, function and neurotransmitter release. Evidence indicates that a low intake of marine omega-3s increases the risk for numerous mental health issues, including Attention Deficit Hyperactivity Disorder (ADHD), autism, bipolar disorder, depression and suicidal ideation. Studies giving supplemental marine omega-3s have shown promise for improving numerous mental health conditions. This paper will review the evidence surrounding marine omega-3s and mental health conditions.

## 1. Introduction

### 1.1. The Rise of Omega-6 Seed Oils and the Fall of Omega-3s in the Diet

Marine omega-3s have been a part of our ancestral diet for millions of years. Estimates indicate that during the Paleolithic era, the intake of the marine omega-3s eicosapentaenoic acid (EPA) and docosahexaenoic acid (DHA) was approximately 660–14,250 mg/day [1,2], compared to around 100–200 mg/day today [3,4]. Moreover, the omega-6/3 ratio has increased from around 4:1 in our hunter-gatherer ancestors to 20:1 today [1,5].

Over the last 100 years, the intake of the omega-6 fat linoleic acid (LA) in the United States has gone from less than 3% of total energy intake to over 7% [6]. The intake of omega-6 has primarily increased due to the consumption of omega-6 rich seed oils, such as soybean, corn and safflower oil, the latter two having an omega-6/3 ratio of approximately 60:1 and 77:1, respectively. During the past century, highly concentrated forms of omega-6 have boosted the omega-6/3 ratio in the Western world from approximately 4:1 or less to approximately 20:1 [5,7]. This ~5-fold increase in the omega-6/3 ratio is reflected by increased LA stored in adipose tissue, which has increased in the United States by approximately 2.5-fold since the 1950s [8]. Furthermore, this rise in omega-6 intake has paralleled the rise in the rates of depression [9]. Importantly, increased omega-6 LA acid intake is associated with an increased risk of depressive and anxiety disorders [10,11]. A high omega-6/3 ratio (especially if above 9/1) is associated with an increased risk for post-partum depression [12]. The protective effects of the parent omega-3 fatty acid alpha-linolenic acid (ALA) against depression is diminished with an increased background intake of LA [13]. This supports the theory that an increase in the omega-6/3 ratio may be a contributing factor in the rise of mood disorders including depression in the Western world.

### 1.2. The Importance of Marine Omega-3s for Brain Function

In order to maintain adequate brain DHA levels in the fetus, non-human primates must consume 0.03% of total energy intake as DHA or 0.45% as ALA [14]. Thus, while ALA can be used to make brain DHA, preformed DHA is much more efficient at this task. This is because there is a low conversion rate of ALA to DHA in non-human primates (estimated to be around 0.2% to 0.57% of ALA converted to DHA) [14]. The conversion rate of ALA to DHA is low because up to 60 to 85% undergoes beta-oxidation [14] to be utilized by tissues for energy (i.e., heart and muscle) or recycled to produce other fatty acids, amino acids (for building proteins) and cholesterol. And approximately 65% of DHA undergoes beta-oxidation. Thus, around two-thirds of ALA and DHA undergo oxidation, whereas around one-third gets stored for energy. This is basically the exact opposite for what occurs with saturated fat (around two-thirds of saturated fat gets stored whereas one-third undergoes beta-oxidation). The liver is better at converting ALA to long-chain fatty acids when intake of ALA is low, however, the data are conflicting regarding ALA conversion in the brain, with some authors believing that the brain is more susceptible to DHA deficiency, particularly since the brain preferentially takes-up LA. Another organ that cannot convert ALA to DHA is the heart, and hence the heart is also susceptible to DHA deficiency.

The optimal conversion rate of ALA to DHA occurs at a dietary omega-6/3 ratio (LA:ALA) ratio of 3:1 to 4:1 [14]. However, since most Western countries consume an omega-6/3 ratio of around 20:1 this decreases the conversion of ALA to DHA. Thus, since most people living in the Western world consume an omega-6/3 ratio above 4:1 this further reduces DHA stores.

Women are able to convert more ALA to EPA/DPA, especially women of childbearing age, due to the greater need of these fatty acids in the growing fetus and greater estrogen levels versus postmenopausal women. The average conversion rate of ALA to EPA is anywhere between 0.2–8.0% but is as high as 21% in younger women [15]. ALA conversion to DHA is only around 0.5%, however young woman may be able to convert as much as 9% of ALA to DHA [15]. The enhanced conversion of ALA to EPA and DHA in woman of childbearing age indicates that EPA and DHA are particularly important to the growing fetus. If this wasn’t the case, then younger women of childbearing age would have no need for better conversion of ALA to EPA/DHA at approximately 10-fold compared to men.

## 2. Early Childhood Development

The International Society for the Study of Fatty Acids and Lipids recommends that women consume 300 mg/day of DHA in pregnancy and lactation. However, the average intake of DHA in pregnant or nursing women is only 60–80 mg/day, which is about 25% of the recommended daily intake [16].

Dietary ALA is primarily converted to DHA in the liver, packaged into triacylglycerol and exported out by the liver within 3 h after its consumption. Once integrated into cellular membranes the omega-3 and omega-6 fatty acids affect the composition and function of membranes, regulate gene expression, and synthesis of eicosanoids, prostaglandins, resolvins and protectins [14,17].

DHA is important for central nervous system (e.g., brain and eye) development and is the most prevalent polyunsaturated fatty acid (PUFA) in the central nervous system. In humans, the accumulation of DHA occurs primarily during the last trimester as well as the first 6–10 months after birth [14]. While the overall diet can alter the fatty acid composition of maternal breast milk, on average, the make-up of breast milk consists of DHA (0.3–0.6%), arachidonic acid (0.4–0.7%), LA (8–17%), and ALA (0.5–1%) [14]. However, optimal breast milk DHA levels may be when DHA makes up 0.8% of total fatty acids (where plasma and red blood cell DHA levels in infants reach their peak) [18].

Children born prematurely miss peak accumulation of DHA from the mother and certain infant formulas may only provide LA and ALA compared to breast milk which also provides DHA. Hence premature babies who are formula fed may be at particular risk of DHA deficiency. A deficiency in DHA is associated with numerous adverse health outcomes such as impaired cognition and visual function, decreased learning ability and altered behavior. DHA in early life is extremely important for visual acuity and only consuming ALA does not provide the same benefits as DHA [14,19].

While the formation of neurons is complete at prenatal stages, gliogenesis, which is the formation of astrocytes (important for neurotransmitter transduction), oligodendrocytes (secrete the myelin that insulates axons allowing electrical signal transduction), and microglial cells (help to remove cellular debris within the central nervous system) does not complete until after birth [14]. Thus, the myelination process does not fully complete until after birth and DHA is extremely important in gliogenesis as it stimulates neurite outgrowth (the outgrowth of dendrites and axons from the neuron), whereas arachidonic acid inhibits this process. Additionally the formation of synapses, which allows one neuron to pass an electrical or chemical signal to another neuron, depends on DHA accumulation in the brain [20]. Hence electrical and chemical/neurotransmitter signaling in the brain depends on having optimal level of DHA. Neurotransmitters in the brain may be affected by low availability of DHA including acetylcholine, dopamine, serotonin, norepinephrine, glutamate and gamma-aminobutyric acid (GABA). Many of these neurotransmitters are extremely important in preventing and treating depression and dementia and this may be why EPA/DHA have benefits in these disease states [20].

Neurons also allow muscle contraction and signal hormone release. Hence, the enhanced formation of neurites and synapses from an optimal DHA intake is important for more than just central nervous system function. Nerve growth cones and synaptosomes incorporate DHA during the formation of synapses suggesting that the mother’s intake of DHA needs to be sufficient for this process to occur at optimal levels [20].

ALA is converted to DHA in the brain, however, consuming pre-formed DHA is more effective at raising DHA levels in glial cells. Glial cells are described as the “glue of the nervous system” as they help to form myelin and provide nutrients to neurons [21,22]. An omega-6/3 (LA to ALA) ratio above 4:1 causes smaller increases in brain DHA content of piglets [23]. Thus, supplemental DHA is likely required to ensure optimal DHA levels in the fetus if the mother’s dietary omega-6/3 (LA:ALA) ratio is above 4:1. Indeed, a high LA intake in the mother, especially early on in the child’s life, may reduce DHA from being incorporated into the brain, which is needed for the formation of neurons and synapses. Pregnant and breastfeeding mothers may need to ensure that their omega-6/3 ratio (LA/ALA) is less than 4:1. In fact, one group of authors concluded “… the optimal LA to ALA ratio for human infants appear to be within the range of 3:1 to 4:1. The cumulative evidence provided by studies in human infants indicate that despite the fact that the ALA-supplemented infant formula contribute efficiently to the maintenance of the omega-3 status in premature newborns, they have a modest impact on DHA levels and that these levels do not reach those observed in breastfed infants” [14].

Human breast milk contains omega-3s and the concentration depends on the dietary omega-3 intake. This may be why breast-fed babies, as compared to babies on formula lacking omega-3s, have a lower incidence of learning disabilities later in life [24,25,26]. A high LA intake in pregnancy is especially hazardous as it lowers EPA/DHA in the umbilical plasma and vein vessel walls and reduces the availability of long-chain omega-3s to the growing fetus [27]. 

Having twins will increase the omega-3 fatty acid need [28]. The omega-3 fatty ALA is considered an essential fatty acid since the body cannot make it and because of this, the omega-3 status of the growing fetus is determined by the intake of the mother. Since the typical western diet is ~20:1 favoring the omega-6 LA, this competes with ALA for conversion to EPA and DHA, making these longer chain omega-3 fatty acids “functionally essential”. Moreover, the high dietary omega-6 further limits omega-3 incorporation into the fetus. 

Preterm neonates have a greater need for omega-3s because the third trimester of pregnancy is when these fatty acids get incorporated into neuronal and retinal tissues [28]. The first 10 months of life are also especially important to ensure appropriate omega-3 status of the newborn. Ensuring appropriate omega-3 supplementation during these important times not only helps with eye and brain development but is also thought to impact cognition, learning, behavior, and reproduction. Importantly, intrauterine omega-3 nutrition early in life likely affects chronic disease susceptibility later in life [28]. 

Adipose tissue is extremely good at storing the omega-6 fat LA but not omega-3 fats (ALA, EPA, or DHA) and hence there is limited storage to draw upon if the intake of omega-3 becomes low compared to omega-6. If DHA is low, the body will begin to synthesize osbond acid (22:5n-6) and hence the ratio between DHA and osbond acid can be a good indicator of DHA status in both the mother and child [28]. In fact, it has been proposed that pregnancy causes DHA deficiency that appears to last even after 6 months postpartum, hence shorter durations between pregnancies, or being pregnant with more than one child, increases the risk of DHA deficiency in both the mother and the fetus [28,29,30].

The hydrogenation of “vegetable” oils leads to trans isomers of unsaturated fats, which interfere with the conversion of parent essential fats to their longer chain fats [31]. Hence our diet high in both omega-6 and trans-fats lowers our omega-3 status. Indeed, trans-fats in chord tissue is associated with lower essential PUFAs, reduced birth weight, and smaller head circumference, all of which are associated with an increased risk of numerous chronic diseases later in life [28]. Thus, not only is it important to reduce the intake of omega-6 LA in pregnancy, but also to lower the intake of trans-fat on top of increasing the intake of omega-3 PUFA (especially DHA). 

Maternal intake of LA, as well as LA content in umbilical plasma phospholipids, is negatively associated with neonatal head circumference [28]. Thus, a higher intake of LA in pregnancy likely leads to neonates with smaller head circumferences and probably lower brain weight. Thus, children born from mothers eating a diet high in LA may have cognitive disadvantages compared to those whose mothers eat less LA. However, the intake of PUFA (when LA is excluded) is positively associated with newborn length, indicating that LA may have a negative effect on the growth of the fetus as well. This might be due to a high maternal LA intake lowering maternal and hence neonatal omega-3 fatty acid status [27].

A high omega-6/3 ratio in the mother may lead to mental developmental illnesses and supplementing preterm infants with marine oils rich in DHA seems to reduce this risk [32]. Randomized trials in humans suggests that supplementing preterm infants with DHA improves intelligence, speeds up visual information processing, and promotes better attention [32,33,34]. Even term infants may have better cognitive performance when given DHA and AA during early postnatal months, showing better problem-solving skills compared to those infants who were not supplemented with the long-chain PUFAs [35].

Breast milk contains DHA but not all baby formulas do. This may be why higher developmental scores at 18 months have been noted in breastfed premature infants versus those who are not. And this may also explain why preterm infants who are given breast milk for 4 weeks or longer have significantly higher intelligence quotient at 7–8 years of age compared to neonates given formula only [36]. Improvements in cognitive development as well as in vocabulary, visuomotor coordination, behavior score, and height and head circumference have been noted [25,26,37]. Breastfeeding, even in term infants, associates with better child cognitive ability and educational achievement and less neurologic abnormalities at 9 years of age [38,39]. All of this suggests that babies who are breast fed are likely to be smarter than those who are bottle fed. Consumption of breast milk or long-chain PUFA-fortified (AA and DHA) baby formula in healthy term infants for 4 months leads to higher developmental quotients compared to infants fed standard baby formulas. [40]. These benefits were associated with a higher red blood cell DHA content [41], whereas a higher red blood cell level of LA correlates negatively with developmental quotient [42]. All of this suggests that a higher postnatal omega-3 intake improves brain and cognitive development, whereas a higher intake of LA likely impairs these outcomes. Postnatal feeding of long-chain marine PUFA (soy oil plus marine oil) to very-low birth weight preterm infants leads to improved visual acuity compared to corn oil or soy oil formulas [43]. Those who were breastfed also had better visual acuity versus those who were bottle-fed. Similar findings of improved visual acuity in preterm infants have been noted in other studies with marine omega-3 supplementation to formula [44,45], benefits which associate with an increase in the red blood cell DHA status. Thus, postnatal marine omega-3s consumption improves retinal function in preterm infants. The post-natal benefits of long-chain omega-3s in term infants on brain and eye development is somewhat controversial, with some studies finding benefits [46,47,48]. Whereas others have not [18,49,50,51,52]. 

In summary, the data suggests that supplemental long-chain omega-3 PUFA intake during pregnancy can have benefits to early childhood development.

## 3. Attention Deficit Hyperactivity Disorder (ADHD)

Attention Deficit Hyperactivity Disorder or ADHD affects anywhere between 4 and 15% of school-aged children in the United States and frequently continues throughout adulthood [53]. ADHD children and adults have been found to have lower levels of long-chain omega-3s in cellular membranes which correlate with behavioral and learning problems such as conduct, hyperactivity-impulsivity, anxiety, temper-tantrums, and sleep difficulties [53]. One double-blind randomized controlled trial in Japan in 40 ADHD-type children noted that providing omega-3 fortified foods (containing around 510 mg DHA and 100 mg EPA/day) improved outcomes on combined teacher and parent ADHD symptom ratings [54]. Another double-blind randomized controlled trial in 50 ADHD-type children showed that an omega-3/evening primrose oil supplement (providing 480 mg DHA, 80 mg EPA, 96 mg GLA and 40 mg arachidonic acid plus 24 mg alpha-tocopherol acetate) significantly improved attention and behavior as well as oppositional defiant disorder compared to placebo (olive oil) [55]. Furthermore, a randomized placebo-controlled trial noted that children and adolescents with ADHD, characterized by inattention and associated neurodevelopmental disorders, responded to an omega-3/6 supplement with meaningful reductions in ADHD symptoms [56]. Yet another randomized controlled trial showed that supplementing children (aged 7–9 years) who were underperforming in reading with 600 mg of DHA (from algal oil) improved parent-rated ADHD-type behavior [57].

In children with impaired visual sustained attention, omega-3 fatty acids esterified to phosphatidylserine (250 mg DHA/EPA plus phosphatidylserine 300 mg/day) noted that 11 out of 18 children became asymptomatic when supplemented with the phosphatidylserine-bound omega-3 supplement, versus 7 out of 21 supplemented with just fish oil, and only 3 out of 21 in the control group [58]. These benefits were corroborated in another study in children with ADHD symptoms supplemented with phosphatidylserine-bound omega-3 supplement (providing 120 mg of DHA/EPA plus 300 mg of phosphatidylserine per day) [59]. Thus, supplementing ADHD children with approximately 120–500 mg of long-chain marine omega-3s/day may provide significant benefits for reducing ADHD symptoms.

## 4. Dyspraxia

Dyspraxia, or developmental coordination disorder (DCD), is a specific impairment of motor function and can affect around 5% of children [53]. Children with this disorder are more likely to have learning, behavioral, and psychosocial issues. A double-blind randomized controlled trial in 117 children (aged 5–12 years) with DCD found that a supplement (providing 732 mg of EPA/DHA and 60 mg of GLA) vs. the group given an olive oil placebo provided significant benefits in reading, spelling, and behavior over 3 months of treatment [60]. Most striking was when children were switched from the olive oil placebo over to the omega-3/evening primrose oil supplement similar benefits were found. Thus, children with dyspraxia or DCD may have improvements in reading, spelling and behavior when provided supplemental marine omega-3s.

## 5. Autistic Spectrum Disorder

Children with autistic spectrum disorder (ASD) have been noted to have low DHA and total omega-3 fatty acids plasma levels [61]. One report found omega-3 fatty acid deficiencies in virtually 100% of ASD cases [53]. And 90% of patients with pervasive developmental disorders (PDD) have been found to have deficient EPA/DHA levels in red blood cell membranes [53]. A double-blind randomized controlled trial in children aged 5–17 diagnosed with ASD found benefit on hyperactivity and stereotypy (the persistent repetition of an act) when given 1.54 g/day of DHA/EPA [62]. And a review paper concluded, “In double-blind, randomized, controlled trials, DHA and EPA combinations have been shown to benefit…autism, dyspraxia, dyslexia, and aggression...” [53]. Thus, supplementing with long-chain omega-3s may help patients with ASD and PDD.

## 6. Mood Disorders

There has been a progressive increase in the prevalence of depression particularly in the Western world after World War II that is unlikely to be entirely attributable to changes in society, diagnostic criteria, or reporting bias. [9,63] Furthermore, as many as 30% to 40% of patients diagnosed with major depressive disorder are considered treatment-resistant [64]. Major depressive disorder is not only a burden on the individual and society but it also contributes to healthcare costs. Indeed, major depressive disorder is estimated to become the second leading cause of disability worldwide by 2020. [65,66] Thus, trying to ascertain and treat the root causes of major depressive disorder is of utmost importance. We propose that the increase in the intake of the omega-6 fatty acid LA from industrial seed oils, as well as the reduction in the intake of long-chain marine omega-3s may be two contributing factors.

The human brain is dynamic with mammalian brains having a synaptic turnover as high as 350% per year, indicating that the mammalian brain is adaptable and has a high plasticity with the ability to make new connections between neurons [53]. One double-blind randomized controlled trial in 33 healthy young patients concluded, “The mood profile was improved after omega-3 with increased vigor and reduced anger, anxiety and depression states” [67]. These benefits occurred in just 35 days of DHA/EPA supplementation (2400 mg of DHA/EPA per day; 800 mg DHA and 1600 mg EPA per day).

## 7. Is Depression Caused by Inflammation in the Brain?

Inflammatory cytokines in the brain can have negative impacts on the central nervous system. One group of authors noted that inflammatory cytokines, “…lower neurotransmitter precursor availability, activate the hypothalamic-pituitary axis, and alter the metabolism of neurotransmitters and neurotransmitter transporter mRNA” [68]. In other words, inflammation may lead to lower neurotransmitter levels in the brain which could potentially predispose people to depression. Additionally, excessive pro-inflammatory cytokines and eicosanoids have been found in patients with depression [68]; with higher levels of monocyte-associated proinflammatory cytokines and chemokines being associated with depression severity [69]. Additionally, certain antidepressants such as tricyclic antidepressants have been thought to work by inhibiting pro-inflammatory cytokine release [70]. Thus, changes in the diet that affect pro-inflammatory cytokine release in the brain, such as omega-3 and omega-6 polyunsaturated fatty acids, have a biological mechanism for affecting our mood [63].

## 8. Higher Intakes of Marine Omega-3s Are Associated with a Lower Risk of Depression

Joseph Hibbeln of the National Institutes of Health found that countries consuming more fish and seafood have lower rates of depression [71]. Other studies have confirmed this finding [72] showing lower risk of suicidal ideation [66]. And better mental health status with higher fish intake [73]. Hibbeln also found an inverse correlation between total seafood intake, as well as DHA in mother’s milk and postpartum depression in 22 countries [74]. Low levels of eicosapentaenoic acid (EPA) and docosahexaenoic acid (DHA) in blood and adipose tissue have been documented in patients with depression [75,76,77].

Lin and colleagues performed a meta-analysis of 14 studies and found lower levels of omega-3 polyunsaturated fatty acids EPA and DHA in those with depression [65]. In patients with recent acute coronary syndromes, those with depression have been noted to have lower levels of EPA and DHA and higher ratios of arachidonic acid/DHA and arachidonic acid/EPA as well as higher omega-6/omega-3 ratios compared to those without depression [78]. Low levels of EPA and/or DHA have also been noted in postpartum depression [79], social anxiety disorder [80], and bipolar disorder. [81]. Thus, omega-3 levels are documented to be lower in red blood cells and plasma of patients with clinical depression with deficits of DHA found in brain tissue of those with major depressive disorder [82]. Moreover, a meta-analysis of 14 studies showed that EPA, DHA and total omega-3 polyunsaturated fatty acids are significantly lower in patients with depression [65]. Thus, the evidence shows that patients with depression have lower levels of omega-3s and higher ratios of omega-6/3.

## 9. The Importance of Long-Chain Omega-3s and Brain Health

Approximately 20% of the dry weight of the brain is made up of polyunsaturated fatty acids and one out of every three fatty acids in the nervous system is a polyunsaturated fatty acid [63]. Docosahexaenoic acid (DHA) is particularly prevalent in the brain and can be retroconverted to eicosapentaenoic acid (EPA) serving as a generator of EPA. Thus, omega-3 polyunsaturated fatty acids are extremely important in brain function and can contribute to disorders of the brain including depression. Both EPA and DHA have been shown to be important in the treatment and prevention of depression, whereas only the latter is a major structural component of neuronal cell membrane phospholipids. 

Marine omega-3s can improve neurotransmitter binding and signaling in the brain by maintaining an optimal membrane fluidity optimizing protein channel function in the lipid bi-layer [63]. Furthermore, neurological and metabolic benefits of consuming marine omega-3s may occur through activating G-protein coupled receptors (GPR40 and GPR120) as well as peroxisome proliferator-activated receptors (PPARs). Indeed, activation of PPAR-alpha can increase hepatic fatty acid oxidation and reduce triglyceride synthesis, PPAR-gamma activation can improve insulin sensitivity in adipose tissue and produce anti-inflammatory effects reducing inflammation and improving insulin sensitivity. GPR120 is highly expressed on adipocytes and inflammatory macrophages and DHA and EPA can promote GPR120-mediated gene activation inhibiting activation of nuclear factor kappa B and reducing inflammation. Furthermore, DHA promotes the translocation of the glucose transporter GLUT4 in adipocytes improving glucose uptake. All of these anti-inflammatory and metabolic effects may have a role in improving brain health. A low dietary intake of marine omega-3s reduces the concentration of omega-3s in cellular membranes, stiffening the membrane and creating a spring-like stress on protein channels which may affect their function. Indeed, low levels of long-chain omega-3 fatty acids in cellular membranes reduces the Na-K-ATPase in nerve terminals, which consumes around half the energy of the brain allowing for nerve transmission and communication [63]. Increasing long-chain omega-3s in the brain may reduce inflammatory cytokines, which may improve neurotransmitter function. There is also a reduction in dopamine and serotonin signaling with omega-3 deficiency in the brain [63]. A deficiency of omega-3 in the brain also reduces synaptic vesicle density in terminals of the hippocampus by 30%, phosphatidylserine levels in the brain by 30–35%, glucose uptake into neurons by 30%, and tyramine-stimulated dopamine release by 90% [63]. The overall consequences of having a long-chain omega-3 deficiency in the brain are summarized in Table 1 and the possible mechanisms for the benefits of omega-3s in depression are summarized in Table 2.

## 10. Clinical Studies Testing Marine Omega-3s in Depression and Other Brain Disorders

In a double-blind, 4-week, parallel-group study in twenty patients with a current diagnosis of major depressive disorder, 2 g of EPA/day improved insomnia, depressed mood and feelings of guilt and worthlessness when added to antidepressant therapy [83]. These benefits were noted in just three weeks after supplementation was initiated. A double-blind placebo controlled study in patents with borderline personality disorder showed that 1 g of EPA/day reduced aggression and severity of depressive symptoms [84]. Another double-blind placebo controlled study found that a total of 6.6 g of omega-3 polyunsaturated fatty acids (providing 3.3 g EPA/DHA twice daily) on top of standard antidepressant therapy in patients with major depressive disorder significantly improved the Hamilton Rating Scale for Depression vs. placebo in just 8 weeks [85]. In patients with treatment-resistant depression, ethyl-EPA given at 1 g/day improved anxiety, depression, lassitude, libido, sleep and suicidal ideation (Table 3 summarizes the key clinical studies) [86]. Moreover, a meta-analysis of 8 randomized controlled trials in patients with depressive symptomatology but no diagnosis of major depressive disorder and 11 randomized controlled trials in patients with major depressive disordered has confirmed that omega-3 polyunsaturated fatty acids are effective in reducing depression severity compared to placebo [87].

Most clinical trials testing marine omega-3 polyunsaturated fatty acids have found improvements in depressive disorders compared to placebo [83,86,89,90,91] with some showing equivalent effectiveness compared to antidepressants such as fluoxetine [88]. Omega-3s have also been found to benefit depression in those with Parkinson’s disease [92] and bipolar disorder [87].

In summary, there are numerous studies and meta-analyses supporting the use of long-chain omega-3s for the prevention and treatment of major depressive disorder and patients with depressive symptoms. Considering that marine omega-3s are safe and well tolerated supplementation with EPA/DHA could be considered in those with depression or who are at an increased risk for developing depression.

In an eight week placebo-controlled, double-blind study, 1 g of ethyl-EPA in 30 female patients with borderline personality disorder was superior to placebo in diminishing aggression as well as the severity of depressive symptoms [84]. Omega-3 polyunsaturated fatty acids may also reduce violence and suicides [93,94]. Moreover, lower levels of EPA have been noted in those with suicide attempts [95]. In patients with recurrent self-harm, supplementing with 2.1 g of EPA/DHA per day improves depression, suicidality and daily stresses [96]. Lastly, supplementation with marine omega-3s has been found to improve depressive symptoms in menopausal women with psychological distress [97], elderly depressed women [98], elderly patients with mild cognitive impairment [99] and juvenile bipolar disorder [100]. Box 1 summarizes the benefits of marine omega-3s in behavior, mood, and other brain disorders.

Box 1 **Behavior, Mood, and Other Brain Disorders that May Benefit from Supplementing with Marine Omega-3s.**

- Attention deficit hyperactivity disorder (ADHD) [101]
- Autism [61]
- Depression [77,102]
- Borderline personality disorder (mood instability and impulsive aggression) [84]
- Schizophrenia [103]
- Hostility [104]
- Anxiety [105]
- Bipolar disorder [106,107]
- Seasonal affective disorder [108]
- Suicidal ideation [93,94,96]

In summary, supplementing with marine omega-3s may benefit behavior, mood, and certain brain disorders. Considering that most of any given population is deficient in EPA and DHA, and that supplementing with marine omega-3s is safe, taking a daily marine omega-3 supplement may be a cost-effective strategy for supporting brain and mood health.

## Figures and Tables

**Table 1:** Consequences of having a long-chain omega-3 fatty acid deficiency in the brain (adapted from Logan) [63].

20% reduction in 5’-nucleotidase activity (decrease membrane fluidity)
30% decrease in synaptic vesicle density in the hippocampus
30% decrease in glucose uptake by neurons
30–35% decrease in phosphatidylserine in brain cortex, brain mitochondria and olfactory bulb
40% decrease in cytochrome oxidase activity
40% reduction in Na-K-ATPase at nerve terminals
Decrease dopamine in vesicle pool, frontal cortex, olfactory bulb
90% decrease in tyramine-stimulated dopamine release from vesicle storage
Decreased dopamine release upon serotonin stimulation
Decreased cerebral microperfusion
Decreased hippocampal CA1 pyramidal neuron cell body size
Decreased vesicular monoamine transporter (VMAT2) which allows dopamine entry/storage in the vesicle)
Decreased pre and post-synaptic dopamine receptor (D2R) in the frontal cortex
Increased serotonin receptor (5HT2) density (indicating reduced serotonin function) similar to that found in those who have committed suicide
Animal studies have found that high levels of omega-3s associate with a 40% increase in frontal cortex dopamine levels including increased binding to the D2 receptor and inhibition of monoamine-oxidase B an enzyme that breaks down dopamine
Reduced nerve growth factor and neurite outgrowth
Decreased amino acid delivery across the blood-brain barrier
Increased proinflammatory cytokines and eicosanoids increased PDE4 activity and a possible reduction in brain-derived neurotrophic factor
Reduced phospholipid biosynthesis and increased phospholipid breakdown (increased brain atrophy)

**Table 2:** Possible mechanisms for the benefits of omega-3s in depression [63].

Improved neuronal membrane stability
Improved serotonin and dopamine transmission
Decreased 5-HT2 receptors and increased D2 receptors in the frontal cortex
Antagonism of arachidonic acid metabolism and metabolites reducing inflammation in the brain
Pro-resolving inflammation/anti-inflammatory effects

**Table 3:** Key Clinical Studies Testing Marine Omega-3s in Depression and Borderline Personality Disorder.

Population	Dose of Omega-3	Outcome
Major Depressive Disorder [83]	2 g of EPA/day	Improved insomnia, depressed mood and feelings of guilt and worthlessness when added to antidepressant therapy
Major Depressive Disorder [85]	3.3 g EPA/DHA twice daily	Significantly improved the Hamilton Rating Scale for Depression vs. placebo in just 8 weeks on top of standard antidepressant therapy
Treatment-resistant Depression [86]	Ethyl-EPA given at 1 g/day	Improved anxiety, depression, lassitude, libido, sleep and suicidal ideation
Major Depressive Disorder [88]	1-g EPA/day	Equally effective in controlling depressive symptoms compared to fluoxetine
Borderline personality disorder [84]	1-g EPA/day	Reduced aggression and severity of depressive symptoms
